# Can Individual and Social Patterns of Resource Use Buffer Animal Populations against Resource Decline?

**DOI:** 10.1371/journal.pone.0053672

**Published:** 2013-01-08

**Authors:** Sam C. Banks, David B. Lindenmayer, Jeff T. Wood, Lachlan McBurney, David Blair, Michaela D. J. Blyton

**Affiliations:** The Fenner School of Environment and Society, The Australian National University, Canberra, Australian Capital Territory, Australia; University of Alberta, Canada

## Abstract

Species in many ecosystems are facing declines of key resources. If we are to understand and predict the effects of resource loss on natural populations, we need to understand whether and how the way animals use resources changes under resource decline. We investigated how the abundance of arboreal marsupials varies in response to a critical resource, hollow-bearing trees. Principally, we asked what mechanisms mediate the relationship between resources and abundance? Do animals use a greater or smaller proportion of the remaining resource, and is there a change in cooperative resource use (den sharing), as the availability of hollow trees declines? Analyses of data from 160 sites surveyed from 1997 to 2007 showed that hollow tree availability was positively associated with abundance of the mountain brushtail possum, the agile antechinus and the greater glider. The abundance of Leadbeater’s possum was primarily influenced by forest age. Notably, the relationship between abundance and hollow tree availability was significantly less than 1∶1 for all species. This was due primarily to a significant increase by all species in the proportional use of hollow-bearing trees where the abundance of this resource was low. The resource-sharing response was weaker and inconsistent among species. Two species, the mountain brushtail possum and the agile antechinus, showed significant but contrasting relationships between the number of animals per occupied tree and hollow tree abundance. The discrepancies between the species can be explained partly by differences in several aspects of the species’ biology, including body size, types of hollows used and social behaviour as it relates to hollow use. Our results show that individual and social aspects of resource use are not always static in response to resource availability and support the need to account for dynamic resource use patterns in predictive models of animal distribution and abundance.

## Introduction

The influence of resource availability on the distribution and abundance of species is a central issue in ecology [1]. It is also a major issue in practical conservation biology because resource decline is a key component of the widespread habitat degradation associated with land use by humans [2], [3]. Many studies have documented declines of species in association with the loss of critical resources, such as hollow-bearing trees that function as shelter resources for many obligate hollow-dwelling arboreal birds and mammals [4], [5]. Often, relationships between resources and animal distribution or abundance are used to make quantitative predictions of how species will respond to scenarios of future resource availability, for instance using population viability analyses or resource selection functions [6], [7], [8].

A key research challenge relating to our ability to predict the responses of animal populations to resource variation is to understand the dynamics of resource use under varying resource availability [9], [10]. Commonly, resource-based models of distribution or abundance assume a static relationship between populations and resources [6]. However, increasing evidence demonstrates that the kinds of resources that are used [11], [12], the frequency with which they are used (or avoided) in relation to their availability (the resource selection function) [13], [14], and the degree of resource sharing (cooperation) [15] can vary with resource availability or other changes such as human disturbance or predation pressure [16], [17]. An understanding of the mechanisms by which animals respond to variation in resource availability is essential if we are to predict how resource variation will affect animal populations [9], [18].

In this study, we investigated whether resource use by hollow-dependent arboreal marsupials varies under resource availability in a semi-natural (*sensu* Franklin & Johnson [19]) forest ecosystem; the tall *Eucalyptus* forests of the Victorian Central Highlands of south-eastern Australia. In these forests, hollow-bearing trees are critical shelter resources for many species, and the availability of hollows is a key conservation issue for a number of arboreal marsupials including the endangered Leadbeater’s Possum (*Gymnobelideus leadbeateri*) [20], [21]. Hollows suitable for most arboreal marsupials typically do not form in mountain ash trees (*Eucalyptus regnans*: the dominant overstorey species) until the trees exceed 190 years of age [22]. There is spatial variation, and an ongoing temporal decline, in hollow availability across this landscape due to different rates of formation and collapse of hollow trees in forest stands of different ages, as well as recent wildfire and logging [23], [24], [25]. Previous work generated projections of temporal declines in the abundance of arboreal marsupials across this landscape by assuming fixed relationships between animal abundance and hollow tree availability [26]. However, adaptive responses in the use of these key resources may mediate the demographic effects of resource variation. Therefore, we tested for two adaptive responses to variation in resource (hollow tree) availability. These were:

### (1) Variation in the Probability of Use of the Hollow Tree Resource

Changes in the use of hollow trees as shelter resources could be manifested in the overall probability of use of the hollow tree resource and in the relative probability of use of different types of shelter resources [11], [27]. We predicted that where hollow trees are scarce, a greater proportion of those trees will be used, and less-preferred kinds (age classes) of trees will be used more often.

### (2) Variation in the Number of Individuals Per Occupied Tree (Resource Cooperation)

Potentially, changes in resource cooperation may either mitigate or exacerbate the demographic effects of resource decline. The evolution of kin-based cooperative behaviour has been documented in response to limitation of territory resources [28], [29]. Such a response (increased resource sharing) could buffer populations against decline in proportion to resource availability. Alternatively, decreasing resource availability can lead to increasing resource competition, with increasing aggression, resource defence and territoriality [30], [31]. Indeed, one of the species studied here, the mountain brushtail possum (*Trichosurus cunninghami*) shared dens less often where dens were less abundant [15], suggesting that social mechanisms can exacerbate the effects of resource decline.

To determine which, if any, of these responses to environmental variation (variation in proportional occupancy and/or resource cooperation) occur, we analysed patterns of abundance, hollow tree occupancy and sharing in four species of arboreal marsupial using a long-term dataset.

## Methods

### Ethics Statement

The field research presented in this paper involved observational animal counts only, and thus did not require an animal ethics permit. The research was conducted in publicly-managed state forests and national parks.

### Study Area and Data Collection

**Figure 1:**
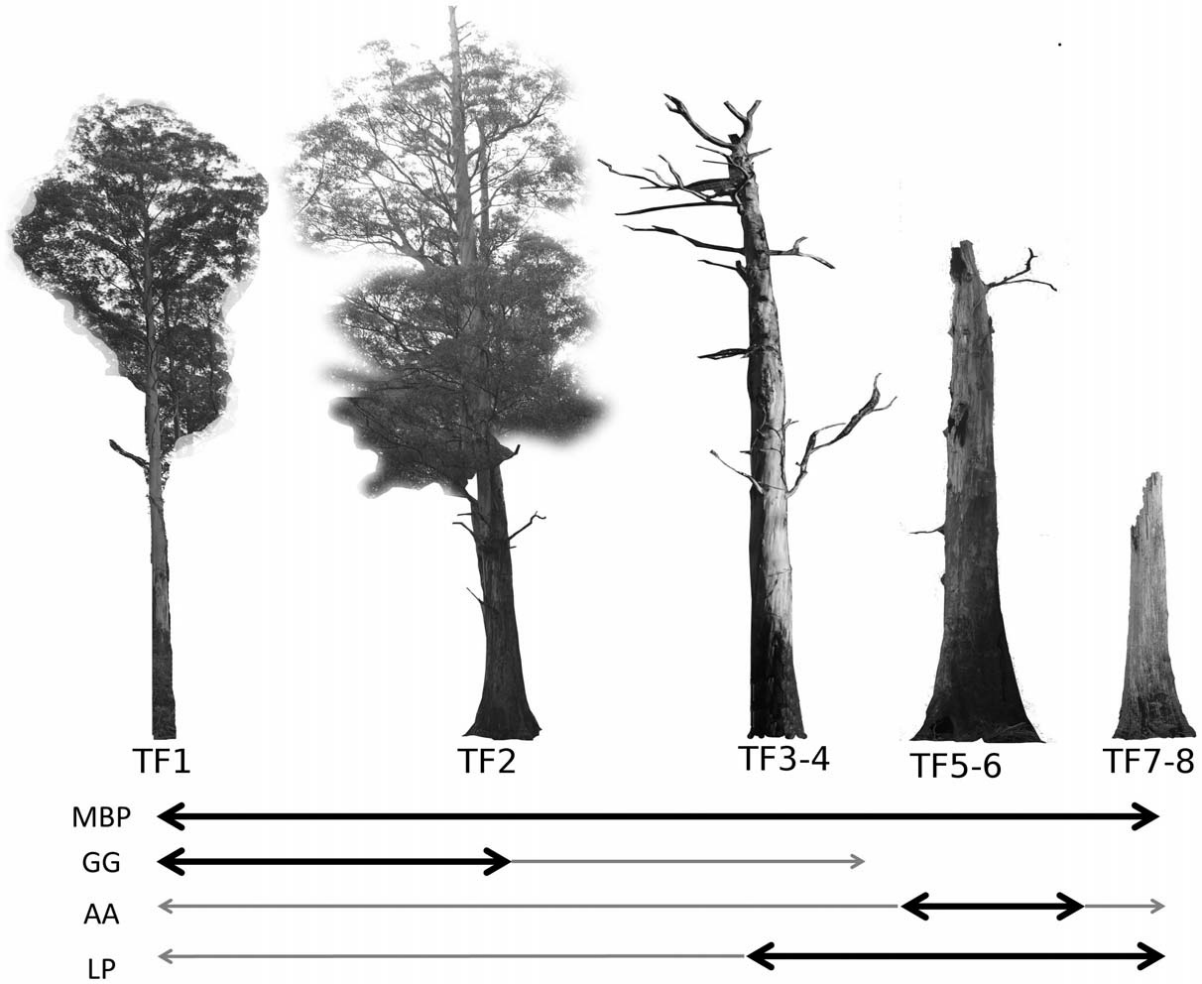
A subset of the decay stages of mountain ash trees used by arboreal marsupials (based on [33], [40]. The dark arrows show the range of tree forms (TF1-8) preferred by each species, including the mountain brushtail possum (MBP), the greater glider (GG), the agile antechinus (AA) and the Leadbeater’s possum (LP). The thinner grey arrows are tree forms used less frequently by each species. Although there is overlap between species in the preferred tree decay stages, the species differ in their specific requirements for hollow size. Mountain ash trees may take up to 150 years from germination to reach the TF1 stage, when suitable hollows for arboreal marsupials first begin to form. Tree form 9 is not shown and represents trees that have completely collapsed. Generally, younger trees (within the range shown) may hollows in the main stem and broken branches, while older trees have hollows in a highly decayed main stem.

We conducted our research in the Victorian Central Highlands of south-eastern Australia, an area covering approximately 60×80 km (37°20′– 37°55′S and 145°30′–146°20′E). The data were collected at 160 one hectare sites that were situated predominantly in mountain ash (*Eucalyptus regnans*) forest. This species is the world’s tallest angiosperm and is the dominant overstorey tree species between 800 m and 1100 m altitude in this area. The number of hollow-bearing trees at each site ranged from one to 31 and were identified on the basis of visual identification of hollows. Each marsupial species studied has specific (and largely non-overlapping) hollow requirements, and the total number of hollow trees per site is likely to be an overestimate of the number of hollow trees available to each species, as not all hollows are suitable for each species. Nevertheless, the type and size of tree hollows (related to their suitability for each species) in a tree is strongly related to its decay stage [32], and we used this as an explanatory covariate in our models. The sites were surveyed repeatedly on an overlapping and rotating sampling design from 1997 to 2007 [33]. During each survey of a site, we counted the number of individuals of each species of arboreal marsupial emerging from every hollow tree on the site for a period of one hour after dusk [33]. All of the species we surveyed are nocturnal. They shelter during daylight hours in tree hollows and typically emerge shortly after dusk to forage. This is the most effective method available for estimating the abundance of each species of arboreal marsupial at a site. We recorded nine species of arboreal marsupial in our surveys [33] but focussed on the four most commonly recorded species for these analyses. These were (1) the mountain brushtail possum (*Trichosurus cunninghami*) a large (2.5–4 kg) nocturnal arboreal marsupial that shelters in large tree hollows; (2) Leadbeater’s possum (*Gymnobelideus leadbeateri*), an endangered small (∼140 g) marsupial with a colonial social system that dens in hollow trees and typically favours small ‘keyhole’ entrances to large hollows inside dead standing mountain ash trees; (3) the greater glider (*Petauroides volans*), a large (1.35 kg) gliding marsupial that feeds exclusively on eucalypt leaves and prefers to den in hollows high in live trees; and (4) the agile antechinus (*Antechinus agilis*) a small (20–40 g) marsupial carnivore that predominantly forages at ground level but dens communally in a range of types of tree hollows. We provide a basic background to the biology of these species in Appendix S1 and a diagrammatic representation of the tree form preferences of each species in Figure 1.

### Data Analysis

We analysed data for each species to answer three questions: (1) Does the number of animals per site vary in proportion to the number of hollow-bearing trees at the site? (2) Does the probability of occupancy of each hollow tree vary in proportion to the number of hollow-bearing trees at the site? (3) Does the number of individuals per occupied tree vary with the number of hollow-bearing trees at the site? We analysed our data using generalised linear mixed models (GLMMs) in Genstat 11 [34]. Our model selection approach was to drop non-significant terms from the ‘full’ model of a small set of candidate explanatory variables. We analysed the data separately for each species because there are no trophic relationships between them, nor are they likely to compete for food or shelter resources (they use different types of hollows [21]), so we had no reason to expect any major effects of one species on another. Indeed, multiple species are commonly detected in the same tree if suitable hollows are available for each. We have no records of multiple species in the same hollow.

#### (1) Does the number of animals per site vary in proportion to the number of hollow-bearing trees at the site?

We used Poisson GLMMs with a logarithmic link function to relate the number of animals of each species per site to candidate explanatory variables. Because each site was surveyed on multiple occasions, year of survey was represented as a random term. Our candidate explanatory variables included the number of hollow bearing trees at that site and the age category of the forest (young regrowth, post 1939 wildfire regrowth, old growth). We included forest age because several important floristic and structural attributes of forest stands vary with age, such as the predominant decay class of hollow trees (Figure 1) and the abundance of *Acacia*, an important food source for species like Leadbeater’s possum. The number of trees per site was analysed both as an untransformed and log-transformed variable. We used these models to answer two questions: (a) Is there an effect of hollow tree abundance on site-level abundance of arboreal marsupials, accounting for potential effects of forest age? (b) If so, is the relationship between arboreal marsupial abundance and hollow tree abundance significantly different to 1∶1. We estimated whether the coefficient differed significantly from 1 by re-fitting the models using log transformed hollow tree abundance as an offset variable.

#### (2) Does the probability of occupancy of each hollow tree vary in proportion to the number of hollow-bearing trees at the site?

**Figure 2:**
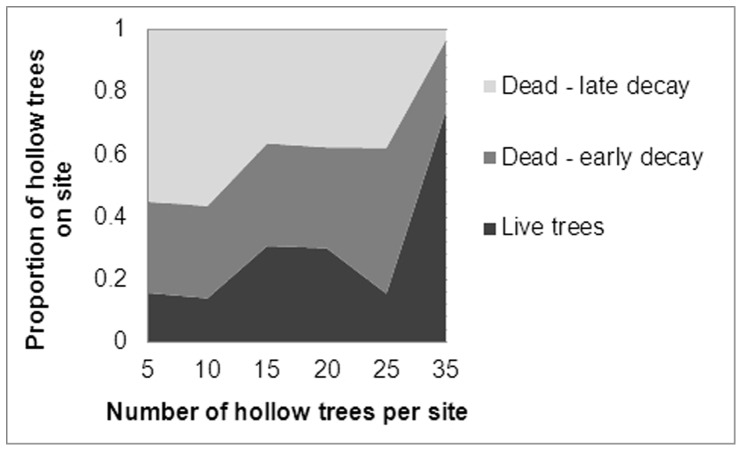
The proportion hollow-bearing trees on each site that are live trees (Tree forms 1–2 in Figure 1), early-decay stage dead trees (Tree forms 3–6) or late-decay stage dead trees (Tree forms 7–8) plotted in relation to the number of hollow-bearing trees per site.

We used binomial GLMMs with a logit link function to analyse the probability of occupancy of each tree by each arboreal marsupial species. Site and year were included in the models as random terms. The candidate explanatory variables (fixed terms) included the number of trees per site (untransformed and log-transformed), forest age category and tree form (Figure 1). Tree form was included because past work indicates that each species has a preference for particular kinds of tree forms [21], and the decay stage of hollow trees that predominate at a site is not independent of the number of trees at that site (Figure 2). For instance, old growth forest stands contain many hollow-bearing trees that are usually alive (Tree forms 1 and 2 in Figure 1). Younger regrowth forests typically contain few hollow trees, and those that are present are often highly decayed ‘legacies’ of an older cohort of trees from before the previous fire (Tree forms 6–8 in Figure 1). Further, the number and type of hollows found in the different tree forms can vary, with the earlier decay classes (Figure 1) often having a number of hollows in broken branches and the later decay classes having fewer, but larger, hollows in a highly decayed main stem [32]. We commenced our analyses with tree form represented as a categorical variable with all nine decay classes (Figure 1). However, after initial exploratory analyses, the tree forms were often condensed to two or three subsets based on the habitat use of each species. For example, for greater gliders we reclassified the tree forms (Figure 1) into a binomial variable distinguishing live trees (Tree forms 1–2) from dead trees (Tree forms 3–8). We included interactions between the number of hollow trees per site and tree form to test for shifts in the kinds of hollow trees selected as dens under variation in den availability (i.e. Is there a ‘relaxation’ of tree form preference as hollow trees become more scarce?).

#### (3) Does the number of individuals per occupied tree vary with the number of hollow-bearing trees at the site?

We used Poisson GLMMs with a logarithmic link function to relate the number of animals of each species observed in occupied trees to the number of trees per site (untransformed and log-transformed), tree form (Figure 1), forest age category and the interaction of these variables. We included tree form to account for potential variation in the type and number of hollows in trees of different decay stages, and forest age class as a broad explanator of variation in structural and floristic attributes of forest stands.

## Results

### (1) Does the Number of Animals Per Site Vary in Proportion to the Number of Hollow-bearing Trees at the Site?

**Figure 3:**
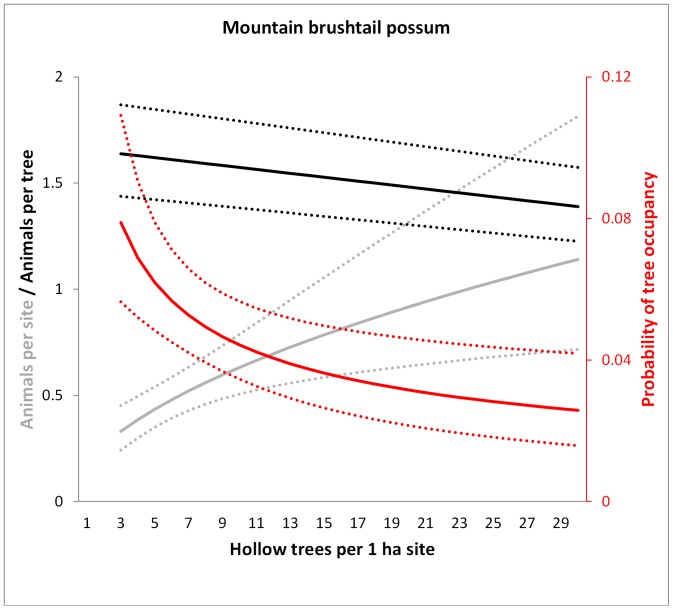
Model predictions for the mountain brushtail possum of the number of animals per site (grey), the probability of occupancy per tree (red) and the number of animals per occupied tree (black) in relation to the number of hollow trees per 1 ha site. Dotted lines show 95% confidence intervals. Predictions were averaged over the non-represented variables (e.g. tree form).

**Figure 4:**
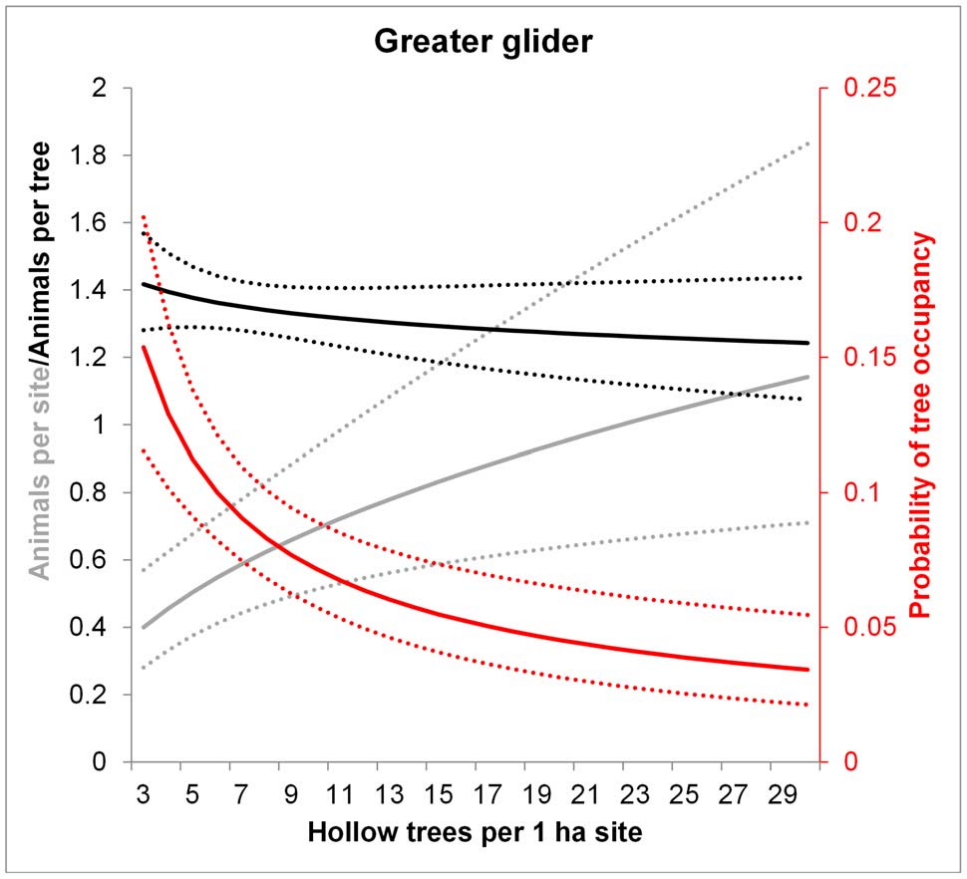
Model predictions for the greater glider of the number of animals per site (grey), the probability of occupancy per tree (red) and the number of animals per occupied tree (black) in relation to the number of hollow trees per 1 ha site. Dotted lines show 95% confidence intervals. Predictions were averaged over the non-represented variables (e.g. tree form). The number of animals per occupied tree (black lines) had a non-significant relationship with hollow tree abundance.

**Figure 5:**
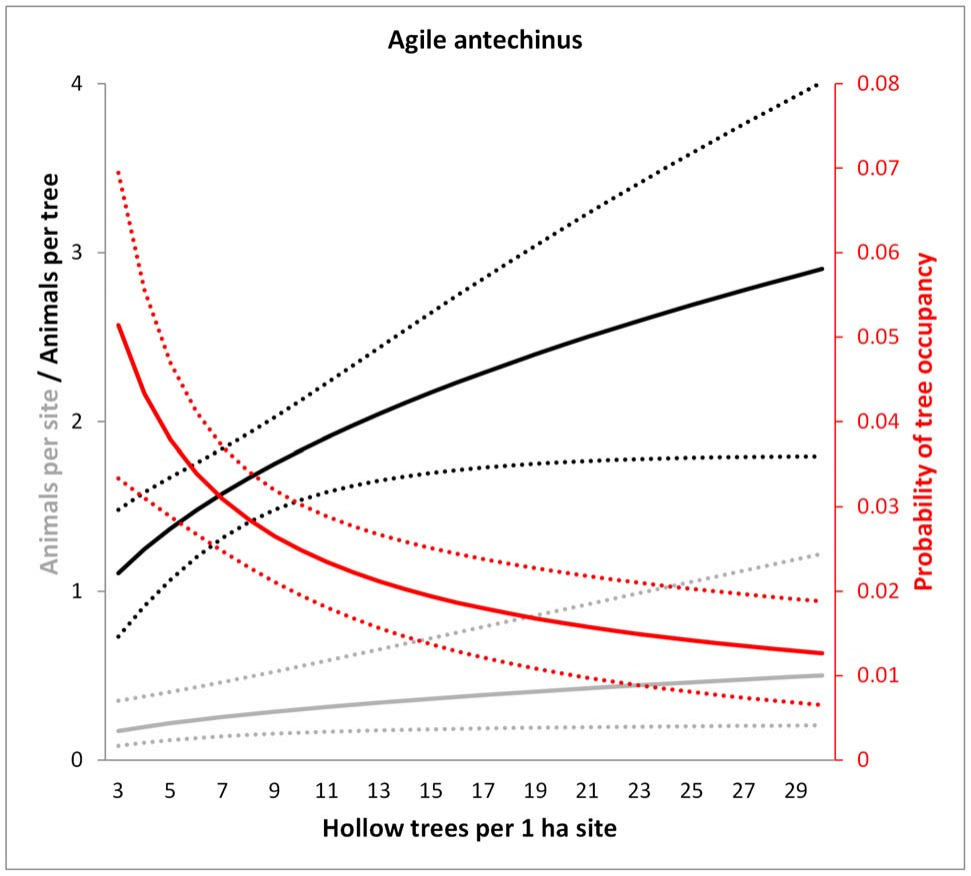
Model predictions for the agile antechinus of the number of animals per site (grey), the probability of occupancy per tree (red) and the number of animals per occupied tree (black) in relation to the number of hollow trees per 1 ha site. Dotted lines show 95% confidence intervals. Predictions were averaged over the non-represented variables (e.g. tree form).

**Figure 6:**
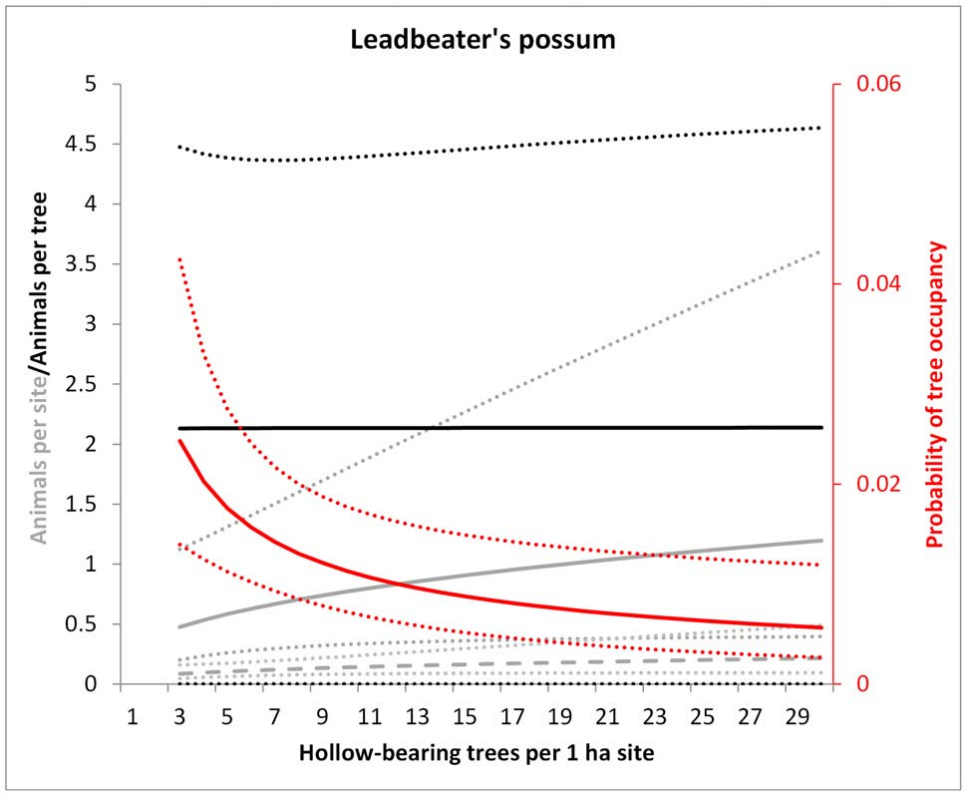
Model predictions for the Leadbeater’s possum of the number of animals per site in 1983 regrowth forest (solid grey line) and older forest (dashed grey line), the probability of occupancy per tree (red) and the number of animals per occupied tree (black) in relation to the number of hollow trees per 1 ha site. Dotted lines show 95% confidence intervals. Predictions were averaged over the non-represented variables (e.g. tree form). All relationships were non-significant except for the occupancy rate (red).

We observed a mean of 2.26 (range 0–21) animals per site (over all species). The most commonly recorded species were the mountain brushtail possum (329 individual records) and the greater glider (328), followed by Leadbeater’s possum (175) and the agile antechinus (160) from 440 site surveys from 1997 to 2007. For three species, the number of individuals recorded per site showed a significant positive relationship with the number of hollow trees (Table 1, Figures 3, 4, and 5). For Leadbeater’s possum, but no other species, we found a significant effect of forest age on site level abundance (this species was most abundant in young regrowth forest that germinated after a 1983 wildfire), but no effect of hollow tree availability (P = 0.082; Table 1, Figure 6).

**Table 1:** Poisson generalised linear mixed models of the effects of hollow tree availability and forest age (non-significant terms were dropped from models) on the abundance of four species of arboreal marsupials.

Species	Term	Coefficient*	S.E.	F	d.f.	P
Greater glider	Year (random effect)	0.100	0.067			
	Site (random effect)	0.576	0.131			
	Constant	−0.497	0.142			
	Ln (Hollow tree count)	0.455	0.129	12.52	184.2	<0.001
Mountain brushtail possum	Year (random effect)	0.017	0.021			
	Site (random effect)	0.721	0.162			
	Constant	−0.559	0.112			
	Ln (Hollow tree count)	0.538	0.148	13.19	176.2	<0.001
Agile antechinus	Year (random effect)	0.576	0.328			
	Site (random effect)	1.351	0.334			
	Constant	−1.261	0.299			
	Ln (Hollow tree count)	0.463	0.230	4.05	163.3	0.046
Leadbeater’s possum	Year (random effect)	0.228	0.141			
	Site (random effect)	1.459	0.348			
	Constant	−2.109	0.239			
	Forest age (Post 1983 growth)	1.71	0.409	17.49	69.4	<0.001
	Ln (Hollow tree count)	0.399	0.228	3.05	193.8	0.082

* Or variance component estimate for random terms.

We were interested in determining whether the relationship between tree hollow abundance and animal abundance differed significantly from 1∶1 and tested this by re-fitting the models using log-transformed hollow tree abundance as an offset variable. The coefficients were significantly less than 1 for all species (P<0.001).

### (2) Does the Probability of Occupancy of each Hollow Tree Vary in Proportion to the Number of Hollow-bearing Trees at the Site?

The average probability of occupancy of a hollow tree was 0.067 (±0.249 s.d.) for the mountain brushtail possum, 0.070 (±0.256) for the greater glider, 0.023 (±0.151) for Leadbeater’s possum and 0.027 (±0.163) for the agile antechinus, over 3466 surveys of individual trees (trees × sites × nights surveyed. For all species, the probability of observing at least one individual emerging from a hollow tree was significantly negatively related to the number of hollow trees at the site (Table 2, Figures 3, 4, 5, and 6). This suggests that a greater proportion of the hollow trees are occupied when there are fewer hollow trees at a site. These relationships were not significantly affected by forest age for any species.

**Table 2:** Binomial generalised linear mixed models of the probability of a hollow tree being occupied by four species of arboreal marsupial.

Species	Term	Coefficient*	S.E.	F	d.f.	P
Greater glider	Year (random effect)	0.137	0.093			
	Site (random effect)	0.588	0.152			
	Constant	−2.570	0.163			
	Ln (Hollow tree count)	−0.699	0.143	22.19	165.9	<0.001
	Tree form (1–2: live trees)	1.175	0.154	58.31	1549.8	<0.001
Mountain brushtail possum	Year (random effect)	0.023	0.033			
	Site (random effect)	0.593	0.158			
	Constant	−3.092	0.226			
	Ln (Hollow tree count)	−1.033	0.327	11.04	176.9	0.001
	Tree form 2	−2.506	0.447	3.05	2253.4	0.003
	Tree form 3	−3.371	0.284			
	Tree form 4	−2.699	0.267			
	Tree form 5	−2.373	0.210			
	Tree form 6	−2.360	0.179			
	Tree form 7	−2.620	0.170			
	Tree form 8	−3.006	0.360			
Agile antechinus	Year (random effect)	0.286	0.185			
	Site (random effect)	0.880	0.259			
	Constant	−4.560	0.368			
	Ln (Hollow tree count)	−0.666	0.206	10.50	155.5	0.001
	Tree form (3–7: early-mid decay)	1.254	0.255	6.78	1962.4	0.001
	Tree form (8–9: highly decayed)	0.964	0.243			
Leadbeater’s possum	Year (random effect)	0.133	0.104			
	Site (random effect)	1.028	0.297			
	Constant	−4.604	0.302			
	Ln (Hollow tree count)	−0.634	0.216	8.61	173.8	0.004
	Tree form (3+: dead trees)	0.639	0.275	5.39	1627.1	0.020

The presented model were selected by dropping non-significant terms from full models of the effects of the number of hollow trees per site, tree form and forest age. For categorical variables (e.g. Tree form) the significance test results for the variable are presented on the line of the first category.

* Or variance component estimate for random terms.

There were significant preferences in the kinds of trees selected for shelter by each species, indicating selection for specific decay classses. Following exploratory analyses, the tree form categories were grouped according to the preference of each species. This included dead trees (Tree forms 3–8 in Figure 1) for Leadbeater’s possum, which were 1.85 times more likely to be occupied than live trees. For the greater glider, live trees (Tree forms 1–2 in Figure 1) were 2.2 times more likely to be occupied than dead trees. Agile antechinus were significantly more likely to be found in trees of medium decay stage (3.2% probability of detection in Tree forms 3–7 in Figure 1) compared to live trees (1% detection rate) or later-stage dead trees (0.6% detection rate). The mountain brushtail possum was less specific in its tree form preference, but it was most likely to be found in hollow bearing trees of form 2 (9.8% detection rate) as illustrated in Figure 1. We did not identify significant interactions between the number of hollow trees per site and tree form on detected tree occupancy by any species (P>0.05 for the interaction in all cases). This suggests no shifts in the kinds of trees selected for shelter in response to variation in the availability of hollow trees.

### (3) Does the Number of Individuals Per Occupied Tree Vary with the Number of Hollow-bearing Trees at the Site?

We recorded a mean of 1.349 range 0–3) greater gliders, 1.418 mountain brushtail possums (0–3), 1.682 agile antechinus (0–7) and 2.160 (0–7) Leadbeater’s possums from each tree found to be occupied by that species. Two species, the mountain brushtail possum and the agile antechinus, showed significant and contrasting social responses to the number of hollow trees per site (Table 3). We found evidence for greater sharing of hollows trees by mountain brushtail possums as hollow trees became scarcer, and a significant effect of tree form, with live trees of tree form 2 (see Figure 1) typically supporting the greatest number of individuals (predicted mean 1.88). Such trees can contain numerous hollows and are most common in old growth forest stands with many hollow trees (Figure 2) [22]. Thus, the kinds of trees predominating at sites with high den availability effectively increases the number of individuals per occupied tree at such sites, yet there also appears to be a behavioural response in the opposite direction, in that den-sharing increases where hollow trees are scarce.

**Table 3:** Poisson generalised linear mixed models of the number of animals in each occupied hollow tree.

Species	Term	Coefficient*	S.E.	F	d.f.	P
Greater glider	Year (random effect)	0.007	0.007			
	Site (random effect)	0.002	0.010			
	Constant	0.281	0.040			
	Ln (Hollow tree count)	−0.047	0.048	0.96	70.9	0.330
	Tree form (1–2: live trees)	0.008	0.058	0.02	107.2	0.889
Mountain brushtail possum	Year (random effect)	0.008	0.008			
	Site (random effect)	0.012	0.011			
	Constant	0.447	0.092			
	Ln (Hollow tree count)	−0.015	0.007	4.37	93.7	0.039
	Tree form 2	0.592	0.118	2.28	144.0	0.025
	Tree form 3	0.089	0.123			
	Tree form 4	0.403	0.102			
	Tree form 5	0.433	0.082			
	Tree form 6	0.238	0.076			
	Tree form 7	0.296	0.070			
	Tree form 8	0.056	0.166			
Agile antechinus	Year (random effect)	0.036	0.041			
	Site (random effect)	0.072	0.066			
	Constant	0.503	0.103			
	Ln (Hollow tree count)	0.368	0.144	6.51	48.2	0.014
Leadbeater’s possum	Year (random effect)	0.048	0.043			
	Site (random effect)	0.092	0.059			
	Constant	0.758	0.108			
	Tree form (3+: dead trees)	0.321	0.188	2.91	71.0	0.092
	Forest age (Post 1983 growth)	0.035	0.167	0.04	25.4	0.836
	Ln (Hollow tree count)	0.001	0.134	0.00	42.8	0.992

Candidate explanatory variables included the number of hollow trees per site, tree form and forest age class. Non-significant terms were dropped from the full models. For categorical variables (e.g. Tree form) the significance test results for the variable are presented on the line of the first category. For Leadbeater’s possum, we present a model that includes a marginally non-significant interaction between forest age and hollow tree count for comparison with models in tables 2 and 3. Likewise, hollow tree abundance and tree form explained no variation in the number of greater gliders per occupied tree: we present the model for comparison with the site abundance and tree occupancy models.

* Or variance component estimate for random terms.

In contrast to the results for the mountain brushtail possum, we found a greater number of agile antechinus per occupied tree in sites where hollow trees were more abundant (Figure 5). The number of Leadbeater’s possums or greater gliders per occupied tree did not vary significantly with the number of hollow trees per site (Table 3).

## Discussion

### Shelter Resources and Arboreal Marsupial Abundance

Our primary aims were to understand how arboreal marsupials respond to the decline of a critical shelter resource, hollow bearing trees, with a specific focus on the dynamics of occupancy patterns and resource sharing under variation in resource availability. Answering these questions contributes to our understanding of the mechanisms by which animals respond to environmental change, thus improving our ability to predict the demographic effects of resource decline [9]. We found the abundance of three hollow-dependent marsupials to be significantly and positively related to the abundance of hollow-bearing trees. However, site-level abundance all species studied decreased at approximately half the rate expected based on a 1∶1 relationship between hollow trees and animal abundance. The two resource use responses that we documented that contributed to this pattern included variation in the probability of occupancy of each hollow tree and in the number of individuals per occupied tree.

### Variation in Occupancy Rates in Response to Hollow Tree Abundance

Of the two responses that we observed, an increase in the probability of occupancy of each hollow tree was the primary demographic compensatory mechanism against shelter resource decline for all species. Indeed, this response was remarkably consistent across the four species studied (Table 1, Figures 3, 4, 5, and 6). Occupancy rates were typically low when hollow trees were abundant (Figures 3, 4, 5, and 6), although this needs to be interpreted in light of: (1) the fact that each species has distinct hollow requirements, so our total hollow tree count is likely to overestimate hollow availability for any individual species; and (2) behavioural aspects of den use, whereby individuals of these species use multiple den trees (over 20 in the case of the mountain brushtail possum [35]). Nevertheless, occupancy rates increased significantly with declining hollow tree availability. Such negative relationships between proportional use of a high quality (or critical) resource and its availability have been observed in other species. For instance, the frequency of use of pastures (containing abundant forage) by red deer (*Cervus elaphus*) increased with decreasing pasture availability in a mosaic landscape comprised of forest and pasture in southern Norway [36]. This change in probability of use of a critical resource with variation in its availability was one of the key responses that we predicted at the outset of this study.

Another response that we predicted was a shift in the relative preference for different resources in response to variation in *per capita* resource availability [37]. Such responses have been observed by other species. For instance, roe deer (*Capreolus capreolus*) showed no change in selection for woodlands in response to their availability. Woodlands provide cover and forage for roe deer, but in agricultural landscapes where woodland cover was low, roe deer increasingly used hedgerows for these purposes [38]. Caerulean warblers (*Dendroica cerulea*) showed a significant shift in the preferred locations of nest sites after major structural habitat changes due to disturbance [39]. This plasticity appeared to confer a degree of demographic resilience to ecological disturbance. In montane ash forests, the marsupials that we studied show preferences for denning in particular decay-classes of tree [4], [21], [40] and we predicted that, in addition to an overall change in the proportion of hollow trees used, the species would show a ‘relaxation’ of tree form selection where hollow trees were less abundant. This was not observed (there were no significant interactions between hollow tree abundance and tree form in tree occupancy models).

It is possible that the structural attributes of the hollows in the different tree decay classes (hollow size, entrance size, elevation, thermal properties) limit flexibility in the different kinds of trees that can be used by each species [20]. However, the lack of a ‘relaxation’ of tree form preference where hollow trees were less abundant was surprising, given that such a relaxation is exactly what was found in a study of one of these species after a recent major fire resulted in the loss of approximately 80% of the hollow trees at one of our sites [23]. Most of the hollow trees that collapsed after that fire were highly decayed dead trees, such that the relative abundance of each tree form was significantly different before and after the fire [23]. However, the ecological context for the variation in hollow tree availability in the dataset analysed here, in which the variation is predominantly spatial (between sites) with a relatively slow temporal change in tree abundance [25], is quite different from the short-term temporal variation caused by fire, where surviving individuals with established home ranges are faced with a dramatically altered resource landscape [23]. Thus, behavioural and demographic variation in response to temporally stable spatial heterogeneity in resource availability may be quite different to behavioural and demographic responses to the rapid loss of a critical resource.

### Resource Sharing

A key aim of this study was to investigate plasticity in resource sharing in response to variation in resource abundance. The availability of, and competition for, resources plays a key role in evolutionary theories relating to social behaviour [28], [29], [41]. There is increasing evidence for social behaviour mediating functional responses to environmental change [42], [43] and for adaptive changes in social behaviour in response to environmental change [44]. This has led to calls for the consideration of social behavioural processes in conservation research and management [45]. Here, we observed variation in resource sharing by two species in response to variation in abundance of hollow trees. However, these responses were smaller than the variation in the resource selection function (i.e. the probability of occupancy of hollow trees) and were highly variable between species. The mountain brushtail possum showed a significant but relatively minor increase in the number of individuals sharing each occupied tree as hollow tree availability declined. The agile antechinus showed a stronger pattern in the opposite direction, while the greater glider and Leadbeater’s possum showed no significant responses.

In the case of the mountain brushtail possum, the demographic consequence of increased den resource sharing in sites with fewer hollow trees was a minor buffering of animal abundance against resource decline. The development of cooperative behaviour of various types in response to a *per capita* decline in resource availability has been observed in other natural systems, including in several bird species [28], [29]. In line with those studies, our results also suggest increased resource cooperation with decreased *per capita* resource availability. Such social responses to resource decline could potentially be an important demographic buffer to otherwise negative environmental changes. However, this is an area that has not been studied extensively in a conservation context. Even within the same study region (and species), different studies have revealed contradictory patterns in different populations. Mountain brushtail possums at Cambarville (within the broader region studied here) shared dens less often and used fewer trees where hollow trees were scarce [15], [23], [46]. These results contradict the present findings and are consistent with predictions from theoretical and empirical work suggesting that resource defence behaviour and intolerance of other individuals develops under resource competition [30]. Potentially, research into kin selection, the scale of individual resource use and resource cooperation, and the scale of heterogeneity in resource availability, may shed light on the discrepancies between these findings [41].

More agile antechinus were observed in each occupied tree on sites with more hollow trees. Most likely, this is a simple consequence of local population size. There are likely to be more animals on sites with more trees because the agile antechinus commonly forages for invertebrates under shed or shedding bark, which is more abundant in old forests than younger forests [47]. The species dens communally in groups of up to 20+ individuals for thermoregulation and pre-mating social interactions {Banks, 2005 #72; [48]. Essentially, there are more individuals available for communal denning in sites with a greater number of hollow trees and they are likely to actively seek out large communal groups. Since communal denning for enhanced thermoregulation is important for this species in cold climates [48], the declining availability of individuals for communal denning with decreasing hollow tree availability may exacerbate the negative effects of hollow tree decline on this species.

### Conclusions and Caveats

We investigated variation in the proportional occupancy and sharing of shelter resources by arboreal marsupials with regard to variation in the abundance of hollow-bearing trees, a critical shelter resource. We found consistent patterns of an increased probability of use of the hollow trees at a given site where there were fewer such trees per site. However, this was not facilitated by a relaxation of preferential selection for certain decay classes of trees by each species. This functional response was the major ‘numerical’ buffer to demographic decline associated with shelter resource loss. An important area for future research in this system will relate to the role of other resources limiting a proportional (1∶1) increase in abundance with hollow tree availability. The probable influence of food resource limitation was apparent in our data for Leadbeater’s possum. For this species, abundance was associated with forest type but not the number of hollow bearing trees. Leadbeater’s possum was most abundant in young regrowth forest. Key habitat requirements for this species include hollow-bearing trees and an understorey of *Acacia* trees for foraging [4], [49]. Several *Acacia* species regenerate rapidly after disturbances such as fires in these forests. Because these regenerating forest stands often contain a number of highly decayed dead trees (the preferred tree form for Leadbeater’s possum: see Figure 1 and Table 2) and high *Acacia* availability, they are likely to be ideal habitat for this species. In contrast, old growth forests contain abundant hollow trees but little *Acacia* understorey [47], such that food limitation is likely to play a larger role than hollow tree availability in the distribution and abundance of this species in such forest stands.

The different social responses that we observed under variation in hollow tree abundance suggest that many aspects of a species’ biology influence the potential for social plasticity in response to variation in resource availability. In this system, variation in other resources such as food, social aspects of the use of the focal resource (hollow bearing trees) and simple physical considerations (e.g. How many animals can fit in a hollow?) are likely to have played important roles. Sociobiology is a relatively new area of research in conservation biology [44], [45], [50]. However, it has a strong foundation in evolutionary ecological research [41], [51] and has the potential to better inform our understanding of the responses of animals to environmental change.

## Supporting Information

Appendix S1 **Basic biology of the four study species.**
(DOCX)
