# Supplementary material for: Can Individual and Social Patterns of Resource Use Buffer Animal Populations against Resource Decline?
Source: PLoS One. 2013 Jan 8;8(1):e53672. doi: 10.1371/journal.pone.0053672 (PMC3539978; doi:10.1371/journal.pone.0053672)
Supplement: Appendix S1 — Basic biology of the four study species. (DOCX) [file pone.0053672.s001.docx]

**Supplementary material for:**

Can individual and social patterns of resource use buffer animal populations against resource decline?

Sam C. Banks, David B. Lindenmayer, Jeff T. Wood, Lachlan McBurney, David Blair and Michaela D.J. Blyton

**Basic biology of the four study species**

Mountain brushtail possum: The mountain brushtail possum (*Trichosurus cunninghami*) is a large (adults approx. 2.5 – 4 kg) and relatively long-lived (~12 years) arboreal marsupial with a broad but predominantly herbivorous diet [[1](#_ENREF_1)]. Females produce a single offspring per year (usually in April in the Victorian Central Highlands) and the rate of monogamy between years by females in this population is approximately 65% [[2](#_ENREF_2)]. There is evidence for behavioural association among some monogamously mating pairs (e.g. commonly co-denning in the same tree [[2](#_ENREF_2),[3](#_ENREF_3),[4](#_ENREF_4)]. The species is nocturnal and shelters in large hollows in live or dead standing trees during the day time [[5](#_ENREF_5),[6](#_ENREF_6)]. It uses large hollows in trees of a range of decay stages.

Leadbeater’s possum: The Leadbeater’s possum (*Gymnobelideus leadbeateri*) is a small possum (adults mean 140 g) with a diet that includes arthropods and plant exudates such as *Acacia* gum [[7](#_ENREF_7)]. The species is considered monogamous and has a colonial social system, with up to 12 individuals sharing a den [[8](#_ENREF_8)]. The species is nocturnal and shelters in tree hollows, typically preferring hollows in later-stage decayed trees with dense vegetation near the hollow entrance. The species prefers areas of forest with large numbers of hollow bearing trees and a dense *Acacia* understorey for foraging [[8](#_ENREF_8)].

Agile antechinus: The agile antechinus is a small (20 – 40 g) carnivorous marsupial with a semelparous life history in which all males die immediately after the breeding season in late

winter (August – September). Females typically produce 6 to 10 off spring and post-weaning dispersal is strongly male-biased [[9](#_ENREF_9),[10](#_ENREF_10)]. A small proportion of females may survive to breed in a second year. Individuals den communally in tree hollows and generally forage at ground level [[11](#_ENREF_11)].

Greater glider: The greater glider (*Petauroides volans*) is the largest of the Australian gliding marsupials (approx. 1.35 kg in our study region) and feeds exclusively on the leaves of eucalypt trees [[8](#_ENREF_8),[12](#_ENREF_12)]. Home range size is about 1 ha [[13](#_ENREF_13)]. The mating system of the species may vary between monogamy and polygamy depending on resource availability, although the species commonly appears to be monogamous [[14](#_ENREF_14)]. One offspring may be produced annually. The greater glider is nocturnal and dens during daylight hours in hollows of large diameter tall eucalypt trees. Typically, live trees with hollows in old growth forest stands are preferred [[15](#_ENREF_15)].

**References**

1. Seebeck JH, Warneke RM, Baxter BJ (1984) Diet of the bobuck, *Trichosurus caninus* (Ogilby) (Marsupialia: Phalangeridae) in a mountain forest in Victoria. In: Smith AP, Hume ID, editors. Possums and Gliders. Sydney: Surrey Beatty and Sons. pp. 145-154.

2. Blyton MDJ, Banks SC, Lindenmayer DB, Peakall R (2012) Using genetic parentage assignment and probability modelling to test the role of local mate availability in mating system variation. Molecular Ecology 21: 572-586.

3. Lindenmayer DB, Welsh A, Donnelly CF (1997) Use of nest trees by the Mountain Brushtail Possum (*Trichosurus caninus*) (Phalangeridae, Marsupialia). III. Spatial configuration and co-occupancy of nest trees. Wildlife Research 24: 661-677.

4. Martin JK, Martin AA (2007) Resource distribution influences mating system in the bobuck (*Trichosurus cunninghami*: Marsupialia). Oecologia 154: 227-236.

5. Banks SC, Lindenmayer DB, McBurney L, Blair D, Knight EJ, et al. (2011) Kin selection in den sharing develops under limited availability of tree hollows for a forest marsupial. Proceedings of the Royal Society B-Biological Sciences 278: 2768-2776.

6. Lindenmayer DB, Welsh A, Donnelly CF (1998) The use of nest trees by the Mountain Brushtail Possum (*Trichosurus caninus)* (Phalangeridae : Marsupialia). V. Synthesis of studies. Wildlife Research 25: 627-634.

7. Smith A (1984) Diet of Leadbeaters Possum, Gymnobelideus Leadbeateri (Marsupialia). Wildlife Research 11: 265-273.

8. Lindenmayer DB (1997) Differences in the biology and ecology of arboreal marsupials in forests of southeastern Australia. Journal of Mammalogy 78: 1117-1127.

9. Banks SC, Ward SJ, Lindenmayer DB, Finlayson GR, Lawson SJ, et al. (2005) The effects of habitat fragmentation on the social kin structure and mating system of the agile antechinus, *Antechinus agilis*. Molecular Ecology 14: 1789–1801.

10. Cockburn A, Scott MP, Scotts DJ (1985) Inbreeding Avoidance and Male-Biased Natal Dispersal in Antechinus Spp (Marsupialia, Dasyuridae). Animal Behaviour 33: 908-915.

11. Lazenby-Cohen KA, Cockburn A (1991) Social and foraging components of the home range in *Antechinus stuartii* (Dasyuridae: Marsupialia). Australian Journal of Ecology 16: 301-307.

12. Kavanagh RP, Lambert MJ (1990) Food selection by the Greater Glider, Petauroides volans: is foliar nitrogen a determinant of habitat quality? Australian Wildlife Research 17: 285-299.

13. Henry SR (1984) Social organisation of the Greater Glider (Petauroides volans) in Victoria. In: Smith AP, Hume ID, editors. Possums and gliders. Sydney: Surrey Beatty and Sons. pp. 222-228.

14. Lindenmayer DB (2002) Gliders of Australia. A Natural History. Sydney: UNSW Press.

15. Lindenmayer DB, Wood J, McBurney L, McGregor C, Youngentob K, et al. (2011) How to make a common species rare: a case against conservation complacency. Biological Conservation In press.
